# Cognitive Control as a 5-HT_1A_-Based Domain That Is Disrupted in Major Depressive Disorder

**DOI:** 10.3389/fpsyg.2019.00691

**Published:** 2019-03-29

**Authors:** Scott A. Langenecker, Brian J. Mickey, Peter Eichhammer, Srijan Sen, Kathleen H. Elverman, Susan E. Kennedy, Mary M. Heitzeg, Saulo M. Ribeiro, Tiffany M. Love, David T. Hsu, Robert A. Koeppe, Stanley J. Watson, Huda Akil, David Goldman, Margit Burmeister, Jon-Kar Zubieta

**Affiliations:** ^1^The Molecular & Behavioral Neuroscience Institute, University of Michigan, Ann Arbor, MI, United States; ^2^Department of Psychiatry, University of Michigan, Ann Arbor, MI, United States; ^3^National Institute on Alcohol Abuse and Alcoholism, National Institutes of Health, Bethesda, MD, United States

**Keywords:** positron emission tomography, intermediate cognitive phenotypes, major depressive disorder, serotonin, executive functioning, interference resolution, processing speed

## Abstract

Heterogeneity within Major Depressive Disorder (MDD) has hampered identification of biological markers (e.g., intermediate phenotypes, IPs) that might increase risk for the disorder or reflect closer links to the genes underlying the disease process. The newer characterizations of dimensions of MDD within Research Domain Criteria (RDoC) domains may align well with the goal of defining IPs. We compare a sample of 25 individuals with MDD compared to 29 age and education matched controls in multimodal assessment. The multimodal RDoC assessment included the primary IP biomarker, positron emission tomography (PET) with a selective radiotracer for 5-HT_1A_ [(11C)WAY-100635], as well as event-related functional MRI with a Go/No-go task targeting the Cognitive Control network, neuropsychological assessment of affective perception, negative memory bias and Cognitive Control domains. There was also an exploratory genetic analysis with the serotonin transporter (5-HTTLPR) and monamine oxidase A (MAO-A) genes. In regression analyses, lower 5-HT_1A_ binding potential (BP) in the MDD group was related to diminished engagement of the Cognitive Control network, slowed resolution of interfering cognitive stimuli, one element of Cognitive Control. In contrast, higher/normative levels of 5-HT_1A_ BP in MDD (only) was related to a substantial memory bias toward negative information, but intact resolution of interfering cognitive stimuli and greater engagement of Cognitive Control circuitry. The serotonin transporter risk allele was associated with lower 1a BP and the corresponding imaging and cognitive IPs in MDD. Lowered 5HT 1a BP was present in half of the MDD group relative to the control group. Lowered 5HT 1a BP may represent a subtype including decreased engagement of Cognitive Control network and impaired resolution of interfering cognitive stimuli. Future investigations might link lowered 1a BP to neurobiological pathways and markers, as well as probing subtype-specific treatment targets.

## Introduction

An enduring, but incomplete observation in MDD (Major Depressive Disorder) is of serotonin dysfunction. Serotonin dysfunction is a corollary of the monoamine hypothesis, positing that MDD is associated with relative depletion of monoamines including catecholamines (e.g., dopamine and noradrenaline) and tryptamine (e.g., serotonin) (Wijaya et al., 2018). Parallel and sometimes convergent reports spanning neurochemistry, behavioral pharmacology, neuroimaging and gene have implicated serotonergic dysfunction in MDD. However, while such dysfunction can be thought of as a typical, it is very clearly not a universal characteristic of MDD (Albert and Lemonde, 2004; Kalia, 2005; Surtees et al., 2006). Evidence that serotonin function is relevant in a subset of those with MDD includes a number of different avenues of exploration. First, affective experience and emotional regulation are more dramatically altered after acute tryptophan depletion (ATD), more so in persons with personal or family history of MDD (Rogers et al., 2003; Fusar-Poli et al., 2006; Neumeister et al., 2006; Spring et al., 2007; van der Veen et al., 2007). Second, selective serotonergic reuptake inhibitors (SSRIs) are more effective than placebo in a majority of controlled clinical trials (Rush et al., 2006; Trivedi et al., 2006). Third, functional loci at genes that mediate serotonergic function have been implicated in MDD both alone, and via interaction with early life stress (Hariri et al., 2002, 2006; Sen et al., 2004; Neumeister et al., 2006; Surtees et al., 2006; Gotlib et al., 2008; Mak et al., 2013). Fourth, some of these functional variants in genes (modulating serotonin function) alter brain responses to emotion and brain connectivity (Dannlowski et al., 2008; Kalin et al., 2008; Elton et al., 2014; Wessa and Lois, 2015). These alternations align with a model of how inherited variations contribute to risk for MDD. Fifth, manipulations of serotonin function and/or use of agents with serotonergic effects within animal models can simulate depression and anxiety-like behaviors (Albert and Lemonde, 2004; Bert et al., 2008; Borg, 2008). Sixth, depression is associated with negative cognitive changes including memory and executive function impairments (Burt et al., 1995; Snyder, 2013; Yu et al., 2018), negative affect related to control, success, and rejection (Yeo et al., 2017) and increased negative schema (Stange et al., 2017; Lim et al., 2018). As such, there is continuing pursuit of domains affected in MDD that can be linked to serotonergic function and genes. There is also interest in whether these dimensional features may define a more homogeneous subset for further exploration and targeted treatment. A short review of the relevant links of serotonin dysfunction in MDD and of potential multimodal intermediate phenotypes [IPs (Burmeister et al., 2008; Kalin et al., 2008; Tan et al., 2008; Langenecker et al., 2010; Webb et al., 2016)] is conducted to integrate these separate lines of inquiry.

### Imaging Studies of 5-HT_1A_ Function

Evidence of abnormal 5-HT (5-hydroxytyptamine refers to G protein coupled receptors and ligand-gated ion channels, also known as serotonin receptors) function in MDD is building, including for 5-HT_1A_ specifically (_1A_ is a subtype of 5-HT receptor which is the most widespread 5-HT receptor, including within cortex and medial temporal structures). Past human imaging studies of 5-HT_1A_ binding potential (BP) have focused on areas of binding where serotonin receptors are more densely populated, including the raphe, as well as frontal, cingulate and medial temporal cortices (Marazziti et al., 1994; Oquendo et al., 2003; Parsey et al., 2006, 2010; Drevets et al., 2007; Selvaraj et al., 2017; Kranz et al., 2018). 5-HT_1A_ receptors regulate the firing of 5-HT neurons presynaptically in the raphe nuclei and are expressed postsynaptically in many different cortical and subcortical brain regions (Schlumpf et al., 1987; Kaufman et al., 2015; Zanderigo et al., 2018). In the cortex, there are inhibitory properties of the postsynaptic 5-HT_1A_ receptors (Gross et al., 2002; Borg, 2008), plus regulation of the release of glutamate in subcortical structures (Czyrak et al., 2003). A recent review of concentrations of transporter (5HTT), 5-HT_1A_, and 5-HT_2A_ receptors reflect the fact that 5-HTTs are densely populated in subcortical, pre-synaptic regions, whereas, 5-HT_1A_ and 5-HT_2A_ receptors are more dense in cortical regions (Kranz et al., 2010; Kautzky et al., 2018). These same cortical areas support a number of cognitive and affective processes and both the regions and the processes they support are heavily implicated in MDD (Teasdale and Dent, 1987; Heller and Nitschke, 1998; Hugdahl et al., 2003; Phillips et al., 2003, 2008; Drevets et al., 2007; Langenecker et al., 2007c, 2010, 2014; Porter et al., 2007; Disner et al., 2011).

Positron emission tomography (PET) studies, typically utilizing the selective radiotracer for 5-HT_1A_ receptors, ^[11C]^WAY-100635, have noted lowered levels of 5-HT_1A_ receptor availability (BP_ND_) within these regions in MDD as well as alterations in 5-HT_1A_ availability pre- and post-treatment (Bhagwagar et al., 2004; Drevets et al., 2007; Moses-Kolko et al., 2007; Hirvonen et al., 2008; Kautzky et al., 2017). Lowered 5-HT_1A_ BP in MDD is the general pattern observed; however, utilizing arterial sampling or a cortical reference region can make a significant impact on the direction of effects [higher or lower (Drevets et al., 2007; Parsey et al., 2010)]. As such, careful verification of reference region/marker equivalence between MDD and healthy comparison (HC) groups is important for PET studies with this radiotracer. Lower brainstem SERT BP was reported in an additional study of depressed suicide attempters (Nye et al., 2013). 5-HT_1A_ disruptions have also been reported in high-risk offspring of those with MDD (Milak et al., 2018).

### Animal Models of 5-HT_1A_ in MDD

Given the limited number and variability across human *in vivo* studies, we briefly review the role of 5-HT_1A_ in the pathophysiology of MDD as seen in animal models of depression and human postmortem studies. Animal studies have primarily reported increased 5-HT_1A_ function after chronic SSRI administration (Haddjeri et al., 1998) and increases in anxious and depressive behaviors after 5-HT_1A_ blockade, depletion, or knockout (Olivier et al., 2001;Akil, 2005; Zhang et al., 2006; Richardson et al., 2010). Novel antidepressants including agomelatine and vortioxetine induced modulation of brain-derived neurotrophic factor (BDNF) which is a neurotrophin that serves as a survival factor for neurons (Lu et al., 2018a,b). In a related study, BDNF knock out mice showed a significant attenuation of 5-HT_1A_ receptor function (Hensler et al., 2007). Acute stress results in decreased 5-HT_1A_ mRNA in the hippocampus (Lopez et al., 1999) and those with knockout or blockade demonstrate memory dysfunction (Sarnyai et al., 2000). Animals with 5-HT_1A_ antagonist acute injection into the dorsal raphe show enhanced social defeat behavior (Cooper et al., 2008).

Stress-sensitive cynomolgus monkeys exhibit a reduced number of 5-HT_1A_ receptors in dorsal raphe after stress exposure (Lima et al., 2009). Similarly, exposure to peer-rearing in rhesus monkeys as an early life stressor generally results in lower *in vivo* 5-HT_1A_ receptor concentrations (Spinelli et al., 2009). Likewise, chronic psychosocial stress in tree shrews results in decreased 5-HT_1A_ receptors in prefrontal cortex, hippocampus, and parietal cortex (Flugge, 1995).

### Human Postmortem and Anatomical Studies

Furthermore, in human postmortem studies, lower hippocampal 5-HT_1A_ mRNA is demonstrated in MDD subjects, with death by accident, assault, suicide, or cardiac causes (Lopez et al., 1998) and reduced 5-HT_1A_ receptors in amygdala and hippocampus in suicide completers (Cheetham et al., 1990). Finally, 5-HT_1A_ receptor density is related to gray matter volume cortical thickness in many prefrontal and parietal regions in HCs, but not in MDD (Pillai et al., 2018; Zanderigo et al., 2018). Notably, one recent study used PET binding to subdivide clusters in 5-HT function for anatomical parcellation and alignment with resting state networks (Kautzky et al., 2018). Both 5-HT_1A_ and 5-HT_2A_ demonstrate cortical; distribution and alignment with dorsal attention and frontoparietal networks (clusters 2 and 3), suggesting that is alignment between monamine function and cortical networks.

### Tryptophan Depletion and Effects on Cognitive Control and Related Functions in MDD

A potentially convergent line of study is a possibility that divergent serotonergic function for some individuals with MDD is related to abnormalities in executive function and affective processing – these are broad domains within Research Domain Criteria [RDoC (Cuthbert, 2005)] that may constitute IPs for MDD. Executive functioning domains include conceptual reasoning, inhibitory control, verbal fluency, interference resolution, working memory – components of the Cognitive Control network. Difficulties in these skills are present in MDD (Austin et al., 1999; Channon and Green, 1999; Rogers et al., 2004; Langenecker et al., 2007b; Snyder, 2013) and lead to work-related disability and productivity loss (Lee et al., 2018). Links between these executive function skills (and cognitive control network function) and serotonergic function are conducted by reducing synthesis of 5-HT centrally via ATD (Lamar et al., 2009; Smith et al., 1999). ATD has also been shown to result in disrupted affective processing and networks (Phillips et al., 2003), increasing negative emotional experience and decreases in positive affective experience [Roiser et al., 2007; Spring et al., 2007; van der Veen et al., 2007]. ATD also disrupts social cooperation (Wood et al., 2006). Some affective domains of interest for MDD are enhanced memory for negative information and disrupted accuracy in processing of facial emotions [Gur et al., 1992; Langenecker et al., 2005, 2007b; Hsu et al., 2010].

In summary, there is a distinct possibility that disrupted 5-HT_1A_ receptor mediated mechanisms might translate to affective and cognitive domains of dysfunction for MDD. Multimodal studies, encouraged by RDoC, can address convergence of multiple different assays. Here, we hypothesized that lower 5-HT_1A_ BP in MDD [Hypothesis (Hyp) 1] maybe related to dysfunction in affective (bottom-up, Hyp 2) and executive (top-down, Hyp 3) domains (Langenecker et al., 2010, 2014; Disner et al., 2011). These affective dysfunction domains included negative memory bias, (Bradley et al., 1996; Hsu et al., 2010) and impaired emotion categorization (Gur et al., 1992; Langenecker et al., 2005, 2007b). We used executive dysfunction domains - previously identified factors in individuals with bipolar disease, similar to domains reported in MDD (Rogers et al., 2004; Snyder, 2013). These factors do not align perfectly with the RDoC domains within Cognitive Systems, although we note that the RDoC domains are suggestive and not prescriptive (Insel et al., 2010; Sanislow et al., 2010). The broader goal is to utilize dimensional, factor-driven analysis in studies where these are experimentally advantageous over DSM categories. Here, Cognitive Control subsumes the elements of (1) speed (Verbal Fluency and Processing Speed), (2) speed in the context of distracting or competing stimuli (Processing Speed with Interference Resolution), (3) stopping a prepotent response (e.g., regulation, here Inhibitory Control), and (4) balance of decision making within multistimulus sets and changing rules (Conceptual Reasoning and Set-Shifting) (Langenecker et al., 2010; Ryan et al., 2013).

We further investigated relationships of fMRI BOLD responses during a Cognitive Control task based upon 5-HT_1A_ BP in the MDD sample, including fMRI BOLD responses based upon degree of 5-HT_1A_ BP_ND_ in the MDD sample [Hyp 4 of lowered 5-HT_1A_ BP_ND_ correlated with lowered activation in Cognitive Control region(s)]. Cross-modality comparisons are relatively rare in MDD (multimodal imaging can be simultaneous or on separate days), but they illustrate the value in integrating localization, function, and neurotransmitter density (Kalin et al., 2008; Selvaraj et al., 2017; Hamilton et al., 2018; Kranz et al., 2018; Piel et al., 2018). Analyses were also conducted with the HC group to verify the general or MDD specific nature of these relationships (Hyp 5). Exploratory analyses with genetic variants related to serotonergic function were also conducted (Hyp 6) (Wojnar et al., 2009; Villafuerte et al., 2009; Mak et al., 2013; Kautzky et al., 2017; Norgaard et al., 2017; Piel et al., 2018; Zanderigo et al., 2018).

## Materials and Methods

### Participants

Twenty-nine HC and 25 patients with MDD were recruited via newspaper advertisements, campus fliers, and word of mouth with Institutional Review Board (IRB)–approved written informed consent consistent with the Declaration of Helsinki at the University of Michigan. Diagnosis was confirmed with the Structured Clinical Interview for Diagnostic and Statistical Manual [DSM-IV (American Psychiatric Association, 1994)]. HC subjects were required to be below 5 and MDD subjects above 15 on the Hamilton Rating Scale for Depression for study entry [HRSD, 17 item scale (Hamilton, 1960)], using conservative thresholds for sensitivity and specificity (Naarding et al., 2002; Romera et al., 2011; Sawamura et al., 2018). The groups did not differ in age, sex, years of formal education, or intellectual ability [(Shipley, 1946), all *p*’s > 0.15, Table 1]. Other evidence of neurological or psychiatric disorders, other than generalized anxiety and/or social/specific phobia, panic disorder in the MDD sample was exclusionary. Cigarette smokers and those with alcohol abuse or who had used illegal drugs in the past 2 years were excluded. Patients with MDD were unmedicated and had been medication-free for a minimum of 6 months for all potentially psychoactive medications (mean 25.7 months, 14 medication naïve).

**Table 1:** Demographic and clinical information for participants with major depressive disorder and matching healthy control adults.

	MDD (*N* = 25)	HC (*N* = 29)
Age	39.7 (11.0)	37.8 (11.8)
Education	15.0 (2.9)	16.1 (2.3)
Shipley estimated IQ	101.8 (13.1)	105.6 (12.1)
Sex	14F, 11 M	18 F, 11M
HDRS-17^∗^	19.0 (2.6)	0.9 (1.4)
Suicide item (0–4)	0.5 (0.9)	–
Neuroticism^∗^	60.3 (11.2)	40.2 (9.6)
Comorbid anxiety Dx	11 (44%)	–

*^∗^Groups differ at *p* < 0.05. Social anxiety (*n* = 7), PTSD (*n* = 2), panic (*n* = 2), generalized anxiety (*n* = 1). HDRS, hamilton depression rating scale. *T*-tests were used to compare groups for age, education, estimated IQ, HDRS, neuroticism. Chi-square was used to compare groups by sex distribution.*

### Overall Procedure

Neuropsychological measures were typically captured within several days after the intake and diagnosis. fMRI and PET were collected on average 9.5 days (*SD* = 36.8) apart for participants. The SD is large because 2 MDD and 2 HC participants discontinued and then restarted the study about 3–4 months apart. 85% of participants completed all evaluations within 1 month.

### PET Scanning and Processing Procedures

**FIGURE 1:**
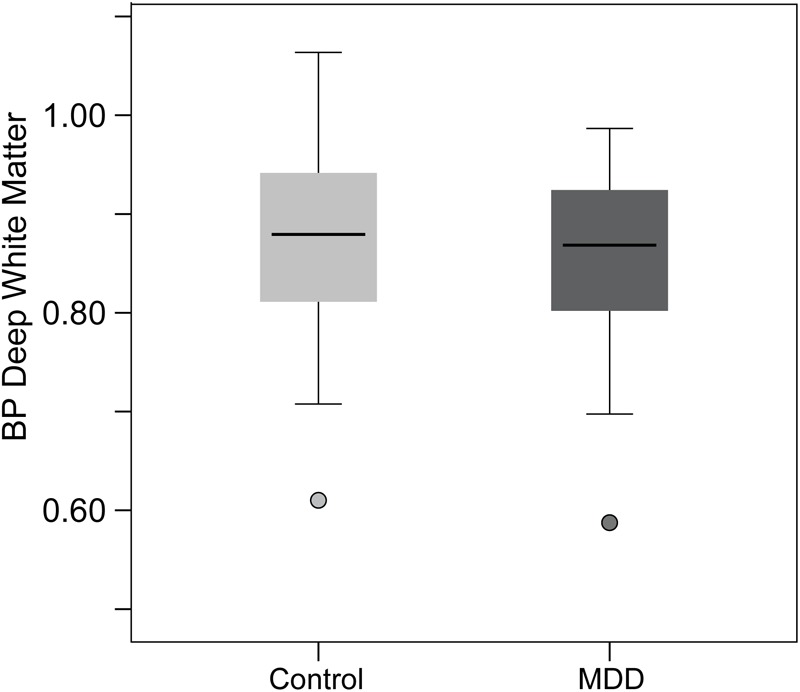
Figure illustrating the 5-HT_1A_ BP values in the deep white matter (DWM) control region. Note that the two individuals (one MDD and one HC) with abnormally high binding potential (BP) in the cerebellar reference region resulted in low values in the DWM region. These two individuals were excluded from all analyses and the MDD and HC did not differ in DWM BP, indicating equivalence in the reference region. Further, no results were changed by including DWM BP as a covariate in analyses.

Positron emission tomography scanning was conducted using [^11^C]Way100635. PET procedures were similar to those described previously (Mickey et al., 2008). PET images were acquired with a Siemens/CTI HR^+^ scanner in three-dimensional mode with septa retracted. [carbonyl- 11C]WAY-100635, a specific 5-HT_1A_ receptor antagonist, was synthesized at high specific activity (Hwang et al., 1999). The tracer was administered as a bolus followed by continuous infusion to more rapidly achieve steady-state conditions. Eighteen scans of increasing duration (0.5–10 min) were acquired over a period of 90 min. Raw PET images were co-registered and smoothed with a Gaussian filter (4 mm FWHM). Smoothed images were transformed voxel-by-voxel into parametric maps of tracer transport (K_1_ ratio) and specific binding [distribution volume ratio (DVR)] using a modified Logan graphical analysis, with bilateral cerebellar white matter (excluding the vermis) as the reference region (Logan et al., 1996). Non-displaceable binding potential (BP_ND_) was defined as BP_ND_ = DVR-1 = k_2_B_max_/K_D_, where B_max_ is the total receptor concentration, K_D_ is the dissociation constant, and k_2_ is the extracellular concentration of tracer (assumed to be a small and constant value) (Stange et al., 2017). Two individuals (one control and one MDD) showed visible binding in the cerebellum and (as a result) anomalously low global BP_ND_ (2.4–3.0 SDs below the mean, illustrated in Figure 1), and were excluded.

Positron emission tomography images were coregistered with MRI images to allow anatomical localization of PET data. Coregistration was accomplished for each subject by alignment of K_1_ images with MRI SPGR images using co-registration within SPM2. MRI data were subsequently transformed into standardized coordinates (International Consortium for Brain Mapping; Montreal Neurological Institute) by linear and non-linear warping, and the resulting transformation matrix was applied to parametric PET images.

Although not central to the current study or hypotheses, we specifically addressed the concern that lowered 5-HT_1A_ BP_ND_ in MDD is a function of differences between HC and MDD in the cerebellar white matter reference region. Without an arterial reference point, we instead added a deep white matter (DWM) ROI within the centrum semiovale for test comparisons between MDD and HC subjects. This technique capitalizes on modeling DWM as a constant in the equation. Without any receptors, DWM would be a constant including noise – the only variable free to vary in the equation is BP within the cerebellar white matter reference region (including noise). There were no significant differences between groups in 5-HT_1A_ BP_ND_ in the DWM of the centrum semiovale [*t*(41) = 0.55, *p* = 0.59]. The reference region BP was equivalent between groups, increasing confidence that effects reported herein are contingent upon inherent regional differences in BP between MDD and HC groups in the regions specified (Figure 1).

### Candidate Affective and Executive Domains Relevant to MDD

The processing speed with interference resolution includes the trail making test, digit symbol substitution test, stroop color-word test, and response time to targets from the parametric go/no-go test. The parametric go/no-go test was programmed in EPrime 2 completed before the scanning session for practice (Langenecker, 2001; Langenecker et al., 2007a,b,c, 2018a; Votruba and Langenecker, 2013). It was also completed during fMRI. There are three levels of difficulty, including a 3 target Go-only condition, and 2 target alternating target Go/No-go condition, and a 2 target alternating target Go/No-go condition. There are 68 “lure” events so that correct and incorrect rejections of lures can be modeled and analyzed separately.

There are also less prominent potential domains/factors, less strongly linked to risk for MDD or BD, comprising Verbal Fluency with Processing Speed, Inhibitory Control, and Conceptual Reasoning and Set Shifting, and tests from these factors have been demonstrated to be dysfunctional in previous studies of MDD (see Langenecker et al., 2009 for a review). Negative Memory Bias (NMB) was calculated as a subtraction of percentage of negative words recognized from the percentage of neutral words recognized from within the Emotion Words task programmed in EPrime 2 (Hsu et al., 2010). In addition to the Negative Memory Bias, we also used performance accuracy in Emotion Classification of faces as potential Affective Processing domains in MDD that would be linked to abnormal 5-HT_1A_ BP_ND_ (Langenecker et al., 2005).

### MRI for Co-registration of PET Images and Collection of fMRI BOLD

One hundred twenty-four high-resolution SPGR axial anatomic images [TE = 5 ms; TR (repetition time) = 24 ms, 45 degree flip angle, NEX (number of excitations) = 2, slice thickness = 1.2 or 1.3 mm, FOV = 24 cm, matrix size = 256 × 256] were performed on each subject with a GE 3T Signa scanner for coregistration of PET images.

The Go/No-go task is a cognitive control task that has been used extensively by our group with fMRI, including in healthy aging, MDD, and bipolar disorder (for review, see Votruba and Langenecker, 2013). The fMRI task includes event-related models for correctly responded “go” events or Hits, correctly rejected “no-go” events or Rejections, and incorrectly responded “no-go” events, or Commissions, modeled with the hemodynamic response function. The steps for processing the data and model building include slice timing, physiological correction, coregistration, normalization, smoothing with a 5 mm FWHM Gaussian filter, and building individual models using SPM2 as described previously (Langenecker et al., 2007c). Contrasts were set up to define activation for Hits, Rejections, and Commissions in a fast event-related model. Imaging parameters include a TR of 2000 ms, FOV of 22 cm, with a 3.0 T GE Signa scanner using a standard radio frequency coil and T2^∗^- weighted pulse sequence. The images were collected using a forward-reverse spiral sequence with 29 axial slices of 4 mm.

### Defining Regions of Significant Effect in 5-HT_1A_, and Low and Normal MDD Groups

Differences between groups in 5-HT_1A_ BP_ND_ will be extracted from regions of significant effects (RSEs). 1st 5-HT_1A_ BP_ND_ levels will be converted to z scores based upon mean and standard deviation of BP_ND_ levels for the HC group for each RSE. Then the z scores will be averaged across all RSEs to create a mean Z Group RSE variable across all post-synaptic 5-HT_1A_ regions that differ between groups. Mean Z group RSE will be used as predictor variable in subsequent analyses with performance and fMRI IPs. We will use mean 5-HT_1A_ BP_ND_ PET results in RSEs to define low and normal 5-HT_1A_ BP_ND_ MDD groups in relation to HC 5-HT_1A_ BP_ND_ in the RSEs. These two MDD groups will then be compared to identify regions for fMRI analyses in the imaging contrasts (Commissions, Correct Rejections, Hits) for the Parametric Go/No-go test.

### Genotyping for 5HTTLPR and MAO-A

In addition, for exploratory purposes, the relative impact for *5-HTTLPR* and *MAO-A* genotype were evaluated, genes with a significant biological relationship with 5-HT_1A_ BP.

#### 5HTTLPR

Genotyping protocols were performed according to Lesch et al. (1996). The *5-HTTLPR* assay discriminates between two functional *5-HTT* promoter alleles, visualized as DNA bands of 528 bp and 484 bp (long and short alleles, l and s, respectively). Genotypes were grouped in accordance with *in vitro* data on a reduced transcriptional activity of the dominating s allele that leads to a decrease in central 5-HT turnover (Bennett et al., 2002). Ten individuals did not have *5-HTTLPR* genotype obtained (6 HC, 4 MDD).

#### MAO-A

Genomic DNA was purified from blood using standard methods. The *MAOA* promoter region that contains the upstream VNTR polymorphism (Sabol et al., 1998) was amplified from 10 ng genomic DNA using the primer sequences: Forward 5 CCCAGGCTGCTCCAGAAACATG-3 and Reverse 5′-GTTCGGGACCTGGGCAGTTGTG-3′. Because of the high GC content in the VNTR region, amplification was performed using Invitrogen’s PlatinumTaq and PCRX. Twenty-three individuals (12 HC, 11 MDD) did not have *MAO-A* genotype obtained.

### Statistical Analyses

First, we identified MDD specific regions of low 5-HT_1A_ BP_ND_ as described in the results section. We compared MDD and HC groups in 5-HT_1A_ BP_ND_ using a combined threshold of *p* < 0.001 and a cluster minimum of 80mm^3^ was used between groups *t*-test in SPM2 (Table 2 and Figure 5). Mean 5-HT_1A_ BP_ND_ was extracted for these Regions of Significant Effects (RSEs) and BP_ND_ was used in group specific linear regressions in SPSS 22 with behavioral performance measures of affective processing (Negative Memory Bias, Emotion Categorization) and executive functioning (Processing Speed with Interference Resolution, Verbal Fluency with Processing Speed, Inhibitory Control, and Conceptual Reasoning and Set Shifting) factors/scores were evaluated as converging, multimodal candidate IPs using MANOVA in SPSS 22. fMRI BOLD activation differences were also investigated in SPM5 factorial model comparisons of normal and low 5-HT_1A_ BP_ND_ MDD groups based upon mean 1A BP_ND_ (with DWM BP as a covariate of no interest). Relationships between fMRI BOLD signal differences were evaluated subsequently with correlations with RDoC Domains scores in SPSS 22. These domains were also evaluated in exploratory analyses for serotonin-related genetic effects using *5-HTTLPR* and *MAO-A*.

**Table 2:** Regions of significantly effect, with lower 5-HT_1A_ BP_ND_ in MDD relative to HC.

Foci	BA	mm^3^	x	y	z	*Z*	*p*
Uncus	20	1344	26	-5	-34	4.38	0.000034
Hippocampus		1536	-25	-13	-20	4.24	0.000053
Parahippocampal	35	1216	-32	-21	-19	3.96	0.00013
Superior temporal	38	960	22	10	-37	4.17	0.000068
Fusiform	37	2624	41	-62	-7	3.78	0.00023
Precuneus	7	832	24	-69	33	3.66	0.00033

*There were no regions where MDD group had greater BP relative to the HC group.*

## Results

### Defining Regions of Low 5-HT_1A_ Binding Potential in the MDD Group

Regions of significant BP differences between HC and MDD groups (HC > MDD) were used to define low 5-HT_1A_ BP_ND_ MDD regions of significant effect (hereafter RSE). The whole brain analyses in MDD vs HC with 5-HT_1A_ BP_ND_ indicated six RSEs of lower 5-HT_1A_ BP_ND_ in the MDD group relative to the HC group. These regions, predominantly temporal, are reported in Table 2 and Figure 5, defining 10–20% reduction in post-synaptic 5-HT_1A_ BP_ND_ in MDD across these regions. There were no regions where MDD group had greater BP_ND_ relative to the HC group. Half of the MDD group was below the 5th percentile of 5-HT_1A_ BP_ND_ for the Z normed average of the RSEs relative to the HC group.

### Impact of Low 5HT1a BP_ND_ on Executive Functioning and Affective Processing IPs in MDD

**FIGURE 2:**
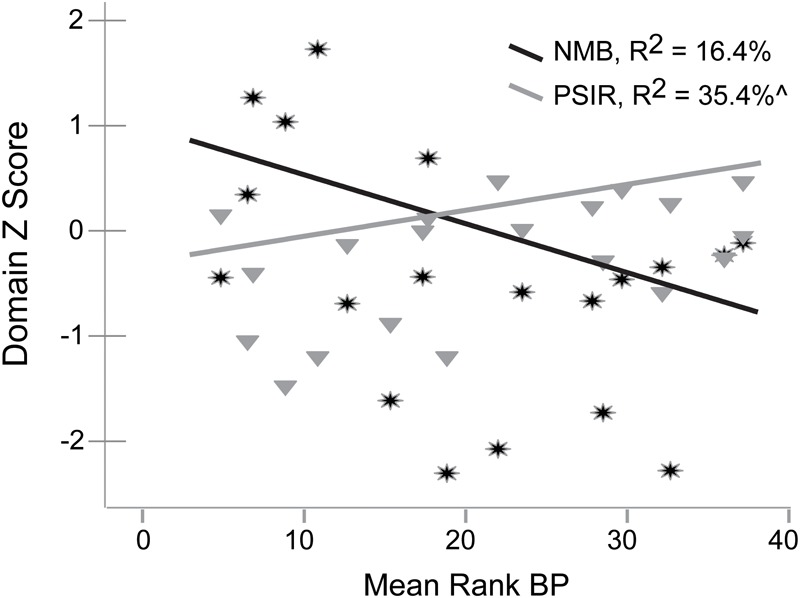
Relationships of Negative Memory Bias (NMB) and Processing Speed with Interference Resolution (PSIR) variables with mean rank 5-HT_1A_ BP in the MDD group.

We investigated the linear relationship of IPs (e.g., Processing Speed with Interference Resolution, Inhibitory Control, and Negative Memory Bias) with mean rank 5-HT_1A_ BP using regression in SPSS 22. Negative Memory Bias accounted for 16.4% (no difference after accounting for age) and Processing Speed with Interference Resolution accounted for 26.3% (35.4% after accounting for age effects) of mean rank BP_ND_ in the MDD group (covarying DWM DVR, *p’*s = 0.074, 0.029, respectively, Figure 2). No other IPs were significantly related to mean rank 5-HT_1A_ BP_ND_ in the MDD group. In the control group, <1% variance in processing speed with interference resolution or negative memory bias was accounted for by mean rank BP_ND_. Negative memory bias and processing speed with interference resolution were non-significantly correlated (*r* = -0.33, *p* = 0.17).

### Lowered Mean 5-HT_1A_ BP in Relation to fMRI BOLD Responses to Hits, Rejections, and Commissions Within the MDD Group

**FIGURE 3:**
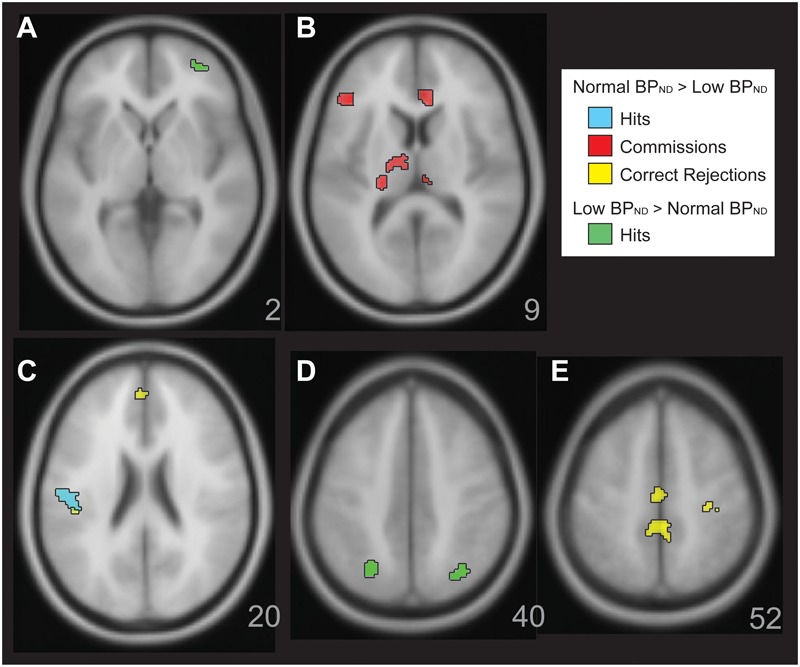
Figure illustrates significant BOLD activation relationships with 5HT-1a BP in the MDD group. There was greater BOLD activation with normal 5-HT_1A_ BP MDD in the Parametric Go/No-go test. This is shown for correct Hits (Panel **C,D**, cyan), for Commissions (Panel **B**, red), Correct Rejections (Panels **C,E**, yellow). There were also a few areas of greater activation for correct Hits with low 5-HT_1A_ BP in the MDD group (Panels **A,D**, green).

It was expected that abnormalities in executive functioning domains based upon mean 5-HT_1A_ BP_ND_ would also be related to BOLD fMRI differences. As this has not been evaluated in published studies, there was no clear expectation of hyper or hypo (our hypothesis) activation during the Cognitive Control task for low vs normal 5-HT_1A_ BP groups. These contrasts in SPM5 included in separate models for BOLD responses during Hits, Correct Rejections, and Commissions. Rejections and Commissions are used to calculate Inhibitory Control, which would be expected to be related to 5-HT_1A_ BP_ND_ based upon relationships illustrated in Figure 3. Processing speed with interference resolution includes response speed to Hits and Conceptual Reasoning with Set Shifting includes Hit accuracy, suggesting that these Hit events should also be related to 5-HT_1A_ BP_ND_. There were some individuals without fMRI scans, some with abnormal DVR in the cerebellum (see section “Materials and Methods”), and some with low IQ, leaving, 17 MDD subjects available for fMRI analyses (divided into normal and low, in a model with 19 HCs). Whole brain analyses were conducted using combined height and extent thresholds with 3dClustSim (*p* < 0.005, *k* > 55, 1000 Monte Carlo simulations, *p* < 0.05 whole brain adjusted).

Within the MDD sample, there was a general pattern of greater activation with increasing mean 5-HT_1A_ BP_ND_. For Correct Rejections (yellow, Figure 3, Panels C, E), this was observed in dorsal anterior cingulate, postcentral gyrus, left posterior insula, and mid cingulate gyrus RSEs. There was increasing activation in rostral anterior cingulate, left inferior frontal gyrus, bilateral dorsal medial thalamus, and pulvinar RSEs with greater mean 5-HT_1A_ BP_ND_ in relation to Commissions. There was greater activation for Hits in a left posterior insula RSE related to mean 5-HT_1A_ BP_ND_ (cyan, Figure 3, Panel C). The exception to this general pattern of increased activation with increasing mean 5-HT_1A_ BP in MDD was observed for Hits in bilateral superior parietal lobule and right anterior inferior frontal gyrus, where there was decreasing activation as mean 5-HT_1A_ BP_ND_ increased (green, Figure 3, Panels A and D).

**FIGURE 4:**
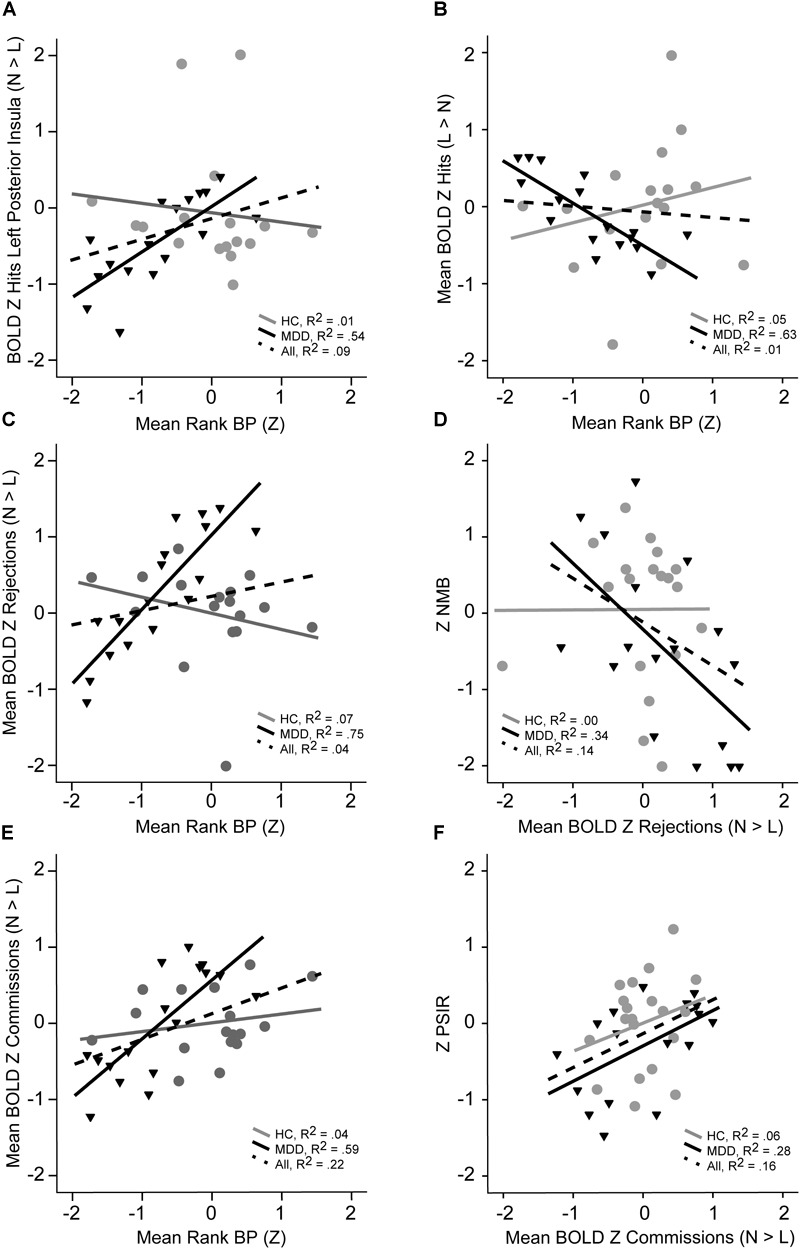
Illustration of linear relationships by group for 5-HT_1A_ BP, fMRI BOLD signal, and neuropsychological performance measures (MDD in black, HC in gray, dashed line for both). Panel **A** shows the relationship between BOLD activation or Hits in left posterior insula with mean 5-HT_1A_ BP rank. Panel **B** illustrates the relationship between mean BOLD signal RSEs for correct Hits that are greater in normal relative to lower 5-HT_1A_ BP in MDD with mean 5-HT_1A_ BP rank. Panel **C** depicts the relationship between mean BOLD for correct rejections and mean 5-HT_1A_ BP rank. The relationship between mean BOLD for correct rejections and NMB in shown in Panel **D**. Panel **E** illustrates the mean BOLD for commission errors with mean 5-HT_1A_ BP rank. The relationship between mean BOLD for commission errors and processing speed with interference resolution is shown in Panel **F**.

We further investigated dimensional, linear links between these multimodal IPs using pairwise correlations between the mean rank 5-HT_1A_ BP_ND_, mean rank for the combined fMRI BOLD RSEs (by condition), and behavioral performance parameters. These fMRI BOLD RSE clusters were combined by condition and group difference for purposes of data reduction, with the resulting mean Z BOLD RSE scores highly correlated with all individual clusters (*r*’s > 0.59 for Commission clusters, *r*’s > 0.68 for Correct Rejection clusters, *r*’s > 0.78 for Hits clusters, *p*’s < 0.001). As illustrated in Figure 4, the mean Z fMRI BOLD RSEs were significantly correlated with mean rank 5-HT_1A_ BP for Hit BOLD RSE Normal > Low (*r* = 0.73, *p* < 0.001, Figure 4, Panel A), Hits mean Z BOLD RSEs Low > Normal (*r* = -0.80, *p* = 0.0001, Panel B), Commissions mean Z BOLD RSEs Normal > Low (*r* = 0.87, *p* = 0.0001, Panel E), and Rejections mean Z BOLD RSEs Normal > Low (*r* = 0.77, *p* = 0.0001, Panel C). PSIR Z score was positively correlated with fMRI BOLD Hit RSE (Normal > Low, *r* = 0.54, *p* = 0.02) and fMRI BOLD Commission mean Z RSEs (Normal > Low, *r* = 0.53, *p* = 0.03, Panel F). Negative Memory Bias Z was significantly positively correlated with fMRI BOLD Hit mean Z RSEs (Low > Normal, *r* = 0.50, *p* = 0.04) and negatively with fMRI BOLD Rejections mean Z RSEs (Normal > Low, *r* = -0.59, *p* = 0.01, Panel D). Information from 19 HCs with all modalities of measurement included – are added to these scatterplots (Figure 4) for comparison. The scatterplots indicate individual level differences in fMRI for Cognitive Control that relate to 5-HT_1A_ BP_ND_ and PSIR.

### Exploratory Analyses of *5-HTTLPR* and *MAO-A* Effects in Executive Functioning and Affective Processing IPs

Next, we expected that the low functioning forms of either the *MAO-A* and *5HTTLPR* genotypes would be associated with poorer performance irrespective of group for candidate genes in exploratory analyses. Given the small sample size, and the relatively weak link between Cognitive/Affective IPs and functional polymorphisms that might impact 5-HT_1A_, the probability of type II error is high. The MANCOVA for *5-HTTLPR* (diagnosis as covariate) was significant for Conceptual Reasoning and Set Shifting [*F*(1,29) = 4.29, *p* = 0.047, *E*^2^ = 0.13] and Processing Speed with Interference Resolution [*F*(1,29) = 5.93, *p* = 0.02, *E*^2^ = 0.17], with poorer performance in *s* allele carriers irrespective of group status. For definition of high and low functioning *MAO-A* genes, the intermediate genotype group of women was placed into the low functioning allele group based upon prior results (Austin et al., 1999) There was a significant effect for genotype on Conceptual Reasoning and Set Shifting [*F*(1,26) = 5.73, *p* = 0.02, *E*^2^ =0 .18] and a trend for Emotion Categorization [*F*(1,26) = 3.35, *p* = 0.08, *E*^2^ = 0.11]. Those with low function alleles for *MAO-A* performed better on Conceptual Reasoning and Set Shifting and marginally worse on Emotion Categorization.

### 5-HT_1A_ BP_ND_ Links to *5-HTTLPR* Genotype and Relationship With PSIR

**FIGURE 5:**
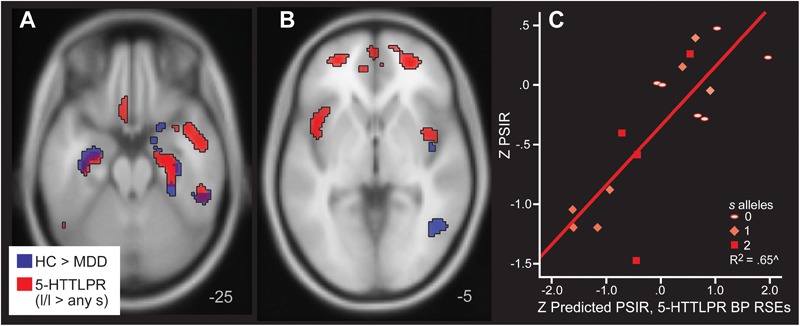
Areas of greater 5-HT_1A_ BP in HC relative to MDD (blue) and in *l* homozygotes for *5-HTTLPR* relative to *s/s or l/s* (red) in Panels **A,B**. Note that the blue clusters are the same as those listed in Table 2, which were used to define the regions of significant effect that defined mean rank BP regressors. They are included for comparison with the *5-HTTLPR* analysis here to show the similarities in location and direction. Panel **C** depicts the actual and predicted PSIR values. The predictions are based upon mean 5-HT_1A_ BP values from the regions of significant effect (RSEs) in the *5-HTTLPR* analysis.

Next, we evaluated specifically the effect of *5-HTLPPR* genotype on 5-HT_1A_ BP_ND_ reductions, covarying for disease group. Group results based upon disease (from Table 2) and genotype are displayed in Figure 5 (Panels A and B). Those with the *5-HTTLPR s* allele, irrespective of disease status, exhibited lower 5-HT_1A_ BP_ND_ in fronto-temporal regions, overlapping with temporal regions that were lower in those with MDD. There were additional frontal regions of lower 5-HT_1A_ BP_ND_ in *s* allele carriers irrespective of diagnosis.

Mean rank order BP based upon *5-HTTLPR* was averaged across all 16 RSEs of greater BP in *l/l* homozygotes relative to the *s* allele carriers. Regression was used to predict Processing Speed with Interference Resolution based upon *5-HTTLPR* and also using mean rank BP from the *5-HTTLPR* RSEs. Fifty-two percent of Processing Speed with Interference Resolution was explained by 5-HT_1A_ BP_ND_ regions with significantly low BP extracted in those with an *s* allele (covarying age and DWM DVR, *B* = 0.79, *p* = 0.001, Figure 5, Panel C). 5-HT_1A_ RSEs defined by MDD vs control and by *5-HTTLPR* were highly correlated (*r* = 0.88, *p* < 0.001) in 5-HT_1A_ BP_ND_. MDD and *5-HTTLPR s* allele were retained as independent variables in this analysis. No other regression models reached significance when using mean rank BP from the *5-HTTLPR* RSEs to predict Negative Memory Bias, Emotion Categorization, Inhibitory Control, or Conceptual Reasoning with Set Shifting.

## Discussion

The present study is the first to link abnormal 5-HT_1A_ BP_ND_ measures in unmedicated, symptomatic patients with MDD to objective performance and imaging markers of illness, in this case interference resolution, a component of Cognitive Control. The separation of interference resolution performance by 5-HT_1A_ levels is marked, with a medium-large effect size. The abnormal 5-HT_1A_ BP_ND_ is also related to fMRI BOLD hypoactivation changes in a Cognitive Control domain during Inhibitory Control, the Parametric Go/No-Go Test. The impact of *5-HTTLPR* genotype upon interference resolution and 5-HT_1A_ is modest and significant. The results follow previous studies showing links between 5-HT_1A_ BP_ND_ values with clinical factors such as anxiety symptoms, treatment outcome, genetics, and sex (Bhagwagar et al., 2004; Drevets et al., 2007; Hirvonen et al., 2008; Miller et al., 2008; Parsey et al., 2010). There is also evidence that lower 5-HT_1A_ levels are present when there are increased depression symptoms in the context of epilepsy, Parkinson disease and in chronic stress without depression (Jovanovic et al., 2008). Using objective, but simpler, performance measures to identify subjects with a higher probability of abnormal 5-HT_1A_ BP_ND_ could have substantial benefits for clinical, genetic and research studies. The executive functioning measures used to derive the processing speed with interference resolution variables are inexpensive to administer and are easily employed in subject recruitment (even clinical) settings (Langenecker et al., 2007b; Dawson et al., 2017). These measures could be used to select individuals for treatments or research protocols that specifically target 5-HT_1A_ receptor functioning and for subtype-specific pharmacotherapy treatment trials. Analogs of these performance measures are already present in animal models to further aid in strategies for better understanding the neurobiology and genetics of depression and for new treatment development.

In the data presented it was striking that Cognitive Control, and not Negative Memory Bias (inverted effect) or Emotion Categorization, was related to lower 5-HT_1A_ BP_ND_ in MDD. Indeed, recent studies have demonstrated that executive functioning measures, an umbrella domain term that includes Cognitive Control, are perhaps most critical in understanding increased risk for MDD, and are observed in the remitted state, and in family relatives of those with mood disorders (Clark et al., 2005; Bora et al., 2009; Peters et al., 2017). A recent review illustrated how executive dysfunction for those with MDD is substantial and fairly consistent across well-powered studies (Rogers et al., 2004). Some existing literature, although mainly with small N studies, suggest that executive functioning is also a good predictor of treatment response and functioning in MDD (Kampf-Sherf et al., 2004; Taylor et al., 2006; Jaeger et al., 2007; Dawson et al., 2017). Executive functioning also can be used to predict recurrence (Langenecker et al., 2018a) and workplace disability (Lee et al., 2018).

A recent review of 5-HT_1A_ receptor studies suggested that there is a weak relationship between 5-HT_1A_ and cognitive function (Borg, 2008). One of four studies in healthy controls have illustrated a relationship between 5-HT_1A_ BP_ND_ and cognitive performance (Yasuno, 2004). A pilot study in those with Alzheimer’s disease, mild cognitive impairment (MCI), or neither suggested a relationship of decreased 5-HT_1A_ BP_ND_ with poorer MMSE in the entire sample, and with learning and memory in the healthy control and MCI participants (Kepe et al., 2006). Another study suggests that gray matter thickness in key limbic regions is positively associated with 5-HT_1A_ (Kraus et al., 2012). A similar study shows these associations in fronto-limbic regions (Zanderigo et al., 2018). In our control sample we replicate the pattern of non-significant relationships of 5-HT_1A_ BP_ND_ to cognitive and affective measures. However, in MDD, we demonstrate a significant positive relationship of 5-HT_1A_ with Negative Memory Bias and Inhibitory Control, and a significant negative relationship of 5-HT_1A_ with Processing Speed with Interference Resolution.

fMRI BOLD signal changes for correct rejections and errors of commission resulted in hypoactivation in critical regulatory and inhibitory regions for those with lower 5-HT_1A_ BP_ND_. These low BOLD signals were related to Negative Memory Bias and Processing Speed with Interference Resolution. Notably, those MDD with lower 5-HT_1A_ BP_ND_ levels show increasing difficulties with poorer set-shifting and processing speed. In contrast and with an intriguing result, MDD subjects with higher/normative 5-HT_1A_ BP_ND_ levels exhibited significant Negative Memory Bias and increased activation in regulatory regions during successful rejection. As a result, increased need for recruitment in the mid-dorsal and rostral anterior cingulate for successful rejection may reflect poorer Inhibitory Control in those without lower 5-HT_1A_ BP_ND_ levels. This observation is confirmed by performance above normal levels in Inhibitory Control as 5-HT_1A_ BP_ND_ levels decreased. Those with higher/normative5-HT_1A_ BP_ND_ 5-HT_1A_ BP_ND_ levels tended to have worse Negative Memory Bias, Inhibitory Control, and increased BOLD recruitment for successful rejection of prepotent stimuli. Although the sample is quite small, these results reaffirm with other work that there likely many circuits, neurotransmitters, and behaviors associated with subtypes of MDD that are heretofore unclear (Webb et al., 2016; Kling et al., 2018).

Further, *5-HTTLPR* appears to be related to both 5-HT_1A_ BP_ND_ levels, and executive functioning performance, irrespective of illness. Notably, samples of this small size often suffer from difficulty with replication and should be interpreted very cautiously. There are some clues, however, that interference resolution might explain the inconsistent findings in emotion processing studies of *5-HTTLPR* from other studies as well. For example, affective processing and executive regulation can be in dynamic opposition to one another in some contexts, or in the case of psychiatric illness, there may be excessive responses in the former *and* weaker control in the latter (Phillips et al., 2003; Kampf-Sherf et al., 2004; Langenecker et al., 2007c, 2014). Presence of the low functioning alleles of *5-HTTLPR* may reflect a relatively weaker executive functioning system, resulting in a stronger environmental dependence in the development of and execution of emotion regulation (Jacobs et al., 2006; Dannlowski et al., 2008; Lohoff et al., 2014; Piel et al., 2018). In non-stressful environments, this weakness is less likely to result in problematic outcomes, but could still result in excessive responses to negative emotional stimuli (Sen et al., 2004; Surguladze et al., 2008). This pattern of diminished regulation skill in those carrying the short allele may be exaggerated in MDD (Dannlowski et al., 2008). In high demand, high stress, negative environments, there may be greater difficulty in regulating negative emotional responses, and greater difficulty in shifting from one emotional state to another for those with low functioning *5-HTTLPR* alleles (Jacobs et al., 2006; Neumeister et al., 2006; Piel et al., 2018). The lack of regulation or emotional flexibility to environmental demands could then perpetuate depressive symptoms.

Use of screening tools like PSIR measures, with knowledge of convergent results with the 5-HT_1A_ BP_ND_ levels may be one dimensional way of increasing the homogeneity in MDD samples. Such increased homogeneity could lead to more targeted, precision medicine trials. For example, preselecting individuals based upon poor Cognitive Control could lead to a larger percentage with low 5-HT_1A_ BP_ND_, facilitating identification of related biomarkers for treatment. As we already know that weaker CC is a predictor of poor treatment response, greater likelihood of recurrence, these individuals might benefit from different treatment algorithms [e.g., TMS trials (Kampf-Sherf et al., 2004; Januel et al., 2006; Siegle et al., 2006; Langenecker et al., 2007c,d, 2018a,b; Levkovitz et al., 2009; Drysdale et al., 2016; Crane et al., 2017; Dawson et al., 2017; Natania et al., 2018)].

It is also notable that Emotion Categorization accuracy, although related at a trend level to MAO-A genotype, was not related to 5-HT_1A_ BP_ND_ levels or *5-HTTLPR*. More surprisingly, higher mean rank BP was associated with *greater* Negative Memory Bias in MDD. Those with lower 5-HT_1A_ BP_ND_ exhibited no evidence of a Negative Memory Bias, suggesting that using mean rank BP to illustrate dimensional functioning in MDD could be defined in part by the segregation of Affective and Executive Domains. In reality, though, risk for MDD is likely defined by affective dysfunction, executive dysfunction, *or* dysfunction in both systems, and it is unlikely to be so clearly illustrated based upon results from one ligand. Several recent studies reported mixed results in links between serotonin genes and emotion reactivity (Kranz et al., 2018; Piel et al., 2018).

The main limitation of the study is the sample size. We were able to obtain a relatively large sample for a multimodal study, and we were also able to find strong biological links between different measurement modalities. Samples of this size only reliably find large and very large effect sizes. We did obtain robust results consistent with our hypotheses. Another potential limitation of the present study relates to the broader difficulty within the field to agree upon the best reference strategy for calculating 5-HT_1A_ BP_ND_. Although we have shown equivalence in our reference region between groups. There are challenges to the reference region approach that appear to be surmountable by excluding gray matter and vermis, such as in this work (Parsey et al., 2010), and verifying equivalence of the reference region, as we have done. Animal models, especially those of 5-HT_1A_ knockout mice, suggest that there may be lower 5-HT_1A_ availability in MDD and similar states, resulting in decreased serotonergic regulation. Likewise, postmortem data also suggests decreased 5-HT_1A_ mRNA in MDD. These findings are contrasted with others suggesting that higher 5-HT_1A_ in mice can mimic aspects of autism and not anxiety/depression. We contend that the present results strengthen evidence that lower 5-HT_1A_ BP_ND_ is a viable IP in MDD. This is in part upon the clear lack of BP difference in a deep white matter region, the presence of a behavioral performance correlate, links to fMRI BOLD, and link to a genetic marker *5-HTTLPR*. At the very least, the fact that 5-HT_1A_ BP_ND_ is altered in some individuals with MDD is clear. Until the discrepant reference region/correction methods can be resolved, directionality is still contested. Individual studies will have to demonstrate equivalence of reference ranges/structures. Furthermore, newer radioligands such as (Elton et al., 2014) MPFF might have more sensitive and stable properties for investigation of 5-HT_1A_ BP_ND_ with MDD subjects absent these reference region concerns (Lothe et al., 2012).

In addition, fMRI is expensive and requires extensive equipment for set-up and analysis. A lower cost alternative to measure hemoglobin changes during task may be functional near-infrared spectroscopy (fNIRS) (Ho et al., 2016). As a number of the imaging regions identified here were cortical, it is possible that fNIRS could be used less expensively and with a broader range of patients to understand the relationship of hemodynamic changes to lowered 5-HT_1A_ function and cognitive control (McKendrick et al., 2015). Finally, neuropsychological testing, fMRI and PET were typically collected on separate days and locations. The anatomical cross-localization could be off in such instances, and the measurements could be weakened by day-session specific parameters (Selvaraj et al., 2017; Hamilton et al., 2018). As the relationships were quite robust, there may have been additional links that were missed.

5-HT_1A_ function has a number of associations with other chronic diseases that are often comorbid with MDD, including coronary artery disease and obesity (Vickers and Dourish, 2004; Ramage and Villalon, 2008; Quek et al., 2017; Ho et al., 2018), and changes in 5-HT_1A_ function are coassociated with changes in pro-inflammatory cytokines including interleukin-1 beta (IL-1β), IL-17, and tumor necrosis factor – alpha (TNF-α) (Aune et al., 1993; Lu et al., 2017; Ng et al., 2018). These studies suggest that 5-HT_1A_ shares functions that are related to, but extend beyond the phenotype of MDD, which in light of the present study, further confirms a need for homogeneous subsets that can be used to explore specific biological pathways for illness and recovery.

## Conclusion

In conclusion, the present study offers promising new evidence that biomarkers for MDD can be found and objectively measured. The heterogeneity of MDD has been problematic in pursuing these biomarkers, and identification of subtypes of MDD may, in the end, prove to be the most fruitful in linking biomarkers, to phenotypes, to genetic risks, and ultimately to personalized medicine. The phenotypic heterogeneity of MDD, combined with prior attempts at a “one size fits all” biomarker approach to MDD has been one limiting factor in this complex illness. Future work can capitalize upon the relationship between 5-HT_1A_ BP_ND_ abnormalities and executive functioning in MDD. These links could also be pursued more broadly in other psychiatric conditions within the RDoC initiative, as executive functioning disruption is not specific to MDD.

## Ethics Statement

The work was approved by the University of Michigan IRB. Written informed consent was obtained from all participants.

## Author Contributions

SL designed the study, analyzed the data, and wrote and edited the manuscript. BM performed the PET and cognitive analyses, and wrote and edited the manuscript. PE, SK, and TL performed the PET analysis, and wrote and edited the manuscript. SS performed the genetics analysis, and wrote and edited the manuscript. KE performed the analysis, and wrote and edited the manuscript. MH and SR wrote and edited the manuscript. DH performed the fMRI and PET analyses, and wrote and edited the manuscript. RK designed PET, and edited the manuscript. SW and HA designed the study and edited the manuscript. DG performed the genetics, analysis and edited the manuscript. MB designed the study, performed the genetics analysis, and edited the manuscript. J-KZ designed the study, and wrote and edited the manuscript.

## Conflict of Interest Statement

The authors declare that the research was conducted in the absence of any commercial or financial relationships that could be construed as a potential conflict of interest.
